# Multi-Armed Bandits in Brain-Computer Interfaces

**DOI:** 10.3389/fnhum.2022.931085

**Published:** 2022-07-05

**Authors:** Frida Heskebeck, Carolina Bergeling, Bo Bernhardsson

**Affiliations:** ^1^Department of Automatic Control, Lund University, Lund, Sweden; ^2^Department of Mathematics and Natural Sciences, Blekinge Tekniska Högskola, Karlskrona, Sweden

**Keywords:** multi-armed bandit (MAB), Brain-Computer Interface (BCI), reinforcement learning, calibration, real-time optimization

## Abstract

The multi-armed bandit (MAB) problem models a decision-maker that optimizes its actions based on current and acquired new knowledge to maximize its reward. This type of online decision is prominent in many procedures of Brain-Computer Interfaces (BCIs) and MAB has previously been used to investigate, e.g., what mental commands to use to optimize BCI performance. However, MAB optimization in the context of BCI is still relatively unexplored, even though it has the potential to improve BCI performance during both calibration and real-time implementation. Therefore, this review aims to further describe the fruitful area of MABs to the BCI community. The review includes a background on MAB problems and standard solution methods, and interpretations related to BCI systems. Moreover, it includes state-of-the-art concepts of MAB in BCI and suggestions for future research.

## 1. Introduction

The multi-armed bandit (MAB) problem, introduced by Robbins (1952), models an agent (decision-maker) that wishes to optimize its actions with the goal of maximizing the expected reward from these actions. The agent must decide between multiple competing actions based on only partial knowledge of their expected rewards and only gains new knowledge after an action is taken. In other words, the agent has to *explore* the action-space before it has enough knowledge to start to *exploit* the learned best action. The exploration vs. exploitation trade-off is recognized from reinforcement learning, which MABs are one the simplest form of Sutton and Barto (2018). MABs have been applied to many different fields of research, such as healthcare, finance, and recommender systems (Bouneffouf and Rish, 2019).

This paper aims to review the MAB framework for the general Brain-Computer Interfaces (BCIs) community. The exploration vs. exploitation tradeoff exists naturally within the procedures of BCI systems, such as deciding which data or paradigm to utilize for a particular task. It is especially so in the online setting, where properties of different choices might only be partially known but become better understood as more data is gathered. The MAB framework provides a structured approach for designing and analyzing BCI systems.

It is assumed that the reader is familiar with the BCI-field and we refer to, e.g., Nicolas-Alonso and Gomez-Gil (2012) or Nam et al. (2018) for any of the BCI-related nomenclature used in the paper. In Section 2, MABs are introduced as well as the algorithms often used to solve them. Section 3 highlights existing examples of MABs in the context of BCIs, while Section 4 provides suggestions for future research. Finally, some MAB programming packages are listed in Section 5 and the paper is concluded in Section 6.

## 2. Multi-Armed Bandits Theory—A Crash Course

### 2.1. The MAB Problem Formulation

The MAB problem is described as: at each time instant *t*, an agent chooses an action *a*_*t*_ out of *K* possible actions and receives a reward *r*_*a*_*t*__. In a BCI setting, MABs could be used to optimize calibration data collection for motor imagery (MI) experiments as in Fruitet et al. (2012). Then, *t* corresponds to the next time for data collection, *K* to the available MI classes, *a*_*t*_ to the class for the next data collection, and *r*_*a*_*t*__ to the increase of classification accuracy when retraining the classifier with the newly gathered data. The reward for each action is not known beforehand. Moreover, the rewards are governed by some probability distribution. This means that the agent needs to perform an action *a*, often multiple times, in order to gain enough knowledge to accurately estimate or predict the reward *r*_*a*_ (Sutton and Barto, 2018).

The aim in a MAB problem is to design a strategy, or policy ϕ, for the agent on how to choose the actions such that the gain, $G_{\phi }\left(T\right)=𝔼\left[\overset{T}{\underset{t=1}{\sum }}r_{a_{t}}\right]$ is maximized. The policy is based on the agent's gathered knowledge from previous actions. The time horizon *T*, also called the agent's budget, is always finite in practice. However, when theoretically analyzing MAB problems, results for finite and infinite time-horizons, *T* → ∞, exist, summarized and explained in Burtini et al. (2015) and Lattimore and Szepesvári (2020a).

In the original MAB problem the rewards are stationary with a binary distribution; 1 or 0, win or lose, with a probability θ_*a*_ of a win (Robbins, 1952). A beta distribution (see, e.g., Faisal et al., 2020) is often used to describe the distribution of θ_*a*_ (different actions have different beta distributions; Scott, 2010). An estimate of the probability to win with an action, ${\hat{\theta }}_{a}$, can for instance be sampled as $\frac{\alpha_{a}}{\alpha_{a}+\beta_{a}}$ where α_*a*_ and β_*a*_ are the number of wins and losses for that action, respectively. The certainty of the estimate increases with the number of samples.

Another common assumption on the rewards' distribution is Gaussianity, see Faisal et al. (2020) for a definition. The reward can then take any value, not only 0 or 1. Each action has an unknown true mean μ for the reward and a standard deviation σ. Upon receiving a reward, the agent can update the estimated values $\hat{\mu }$ and $\hat{\sigma }$ (Sutton and Barto, 2018). Many other assumptions on the rewards' distributions can be made, and we refer to Lattimore and Szepesvári (2020b) for further information.

The MAB problem can be varied in multiple ways. For instance, the probability distributions of the rewards *r*_*a*_*t*__ can be considered to be stationary or changing over time. The set of possible actions *K* can be fixed or non-fixed. The reward distributions could change depending on contextual information, and the policy of the agent needs not be restricted to one action at a time. Table 1 illustrates the so-called original MAB problem, restless and switching bandits, mortal and sleeping bandits, contextual bandits as well as dueling bandits, some common variants of MAB problems.

**Table 1:** Overview of MAB variants and their characteristics compared to the original MAB problem.

**Multi-armed bandit variant**	**Characteristic**
Original MAB problem	Static reward and fixed set of actions
Restless and switching bandits	Non-static reward
Mortal and sleeping bandits	Set of available actions changes
Contextual bandits	Rewards change based on state of surrounding environment
Dueling bandits	Agent chooses two actions at each time step

### 2.2. Algorithms for Solving MAB Problems

The aim for all algorithms, also called policies, is to balance the exploration vs. exploitation of the actions (Sutton and Barto, 2018). Here, we present the most common algorithms in the context of the original MAB problem formulation. We refer to the survey by Burtini et al. (2015) and the book by Lattimore and Szepesvári (2020a) for other algorithms.

The *regret*, *R*_ϕ_(*T*), is used to evaluate and compare algorithms. It is the difference between the total reward for the best action and the agent's gained reward over the time horizon *T*. In Equation (1), r^*^ is the best achievable reward, i.e., the expected reward for the best action, and *r*_*a*_*t*__ is the agent's received reward at each time step using the policy ϕ. The theoretical (upper) bounds on the regret, meaning the worst-case expected regret after *n* number of plays, are often compared for different policies. If the regret bound is logarithmic, the optimal action is found with the policy. Analysis of the lower bounds on the regret shows the best case for finding the optimal action (Burtini et al., 2015; Lattimore and Szepesvári, 2020b).


(1) $$\begin{array}{l} R_{\phi }\left(T\right)=Tr^{*}-𝔼\left[\overset{T}{\underset{t=1}{\sum }}r_{a_{t}}\right]=Tr^{*}-G_{\phi }\left(T\right) \end{array}$$

#### 2.2.1. Random Policy

In the random policy, the agent takes a random action at each time instance. This policy is often used as a baseline when comparing policies—a policy should not be worse than the random policy.

#### 2.2.2. ϵ-Greedy Policy

The agent gets an initial estimate of each action's reward by performing each action once. In a greedy policy, the agent always chooses the action with the highest estimated reward. This method only exploits and never explores after the initial phase. If the agent's initial reward estimates are off, the policy will be stuck in always choosing a non-optimal action, giving a linear regret growth.

In the ϵ-greedy policy on the other hand, the agent chooses the best action but with an ϵ probability picks a random action (Sutton and Barto, 2018). The occasional random action forces the agent to explore all actions, which helps the agent to better estimate the actions' rewards so the agent can exploit the best action. Though the occasional random action will force the agent to act non-optimally, which is unwanted. Gradually decreasing ϵ over time reduces the probability of such non-optimal actions. Theoretically, such ϵ-decreasing policies can be constructed which guarantee logarithmic bounds on regret (Auer et al., 2002), which is a significant improvement over linear growth.

Another variant of the ϵ-greedy policy is the ϵ-first policy. The agent takes a random action for the first ϵ*T* time steps and picks the action with the highest estimated reward for the remaining (1 − ϵ)*T* steps. This policy has proven to be superior to the ϵ-greedy policy when the time horizon is known and the rewards are stationary (Burtini et al., 2015).

#### 2.2.3. Upper Confidence Bound (UCB)

In the Upper Confidence Bound (UCB) algorithm, the agent looks at the estimated reward plus an extra margin based on the uncertainty of the reward's estimate. The extra margin is calculated from the number of actions that have been taken in total and the number of times that action has been taken. The algorithm for the next action *a*_*t*_ is mathematically described as Equation (2) where ${\hat{r}}_{a}$ is the estimated reward for that action, *t* is the current time step, *n*_*a*_ is the number of times the action has been taken and *c*>0 is a parameter. The UCB algorithm was extensively developed in Auer et al. (2002) and is summarized in Sutton and Barto (2018).


(2) $$\begin{array}{l} a_{t}={\text{argmax}}_{a}\left[{\hat{r}}_{a}+c\sqrt{\frac{\ln t}{n_{a}}}\right] \end{array}$$

The UCB algorithm does not have any assumption on the distribution of the rewards, and its regret is logarithmically bounded, as proven in Auer et al. (2002). There are many variants of the UCB algorithm that cope with non-stationary rewards or contextual information, such as LinUCB, Adapt-Eve, DiscountedUCB, and SlidingWindowUCB (Burtini et al., 2015).

#### 2.2.4. Thompson Sampling

Thompson sampling, first introduced in Thompson (1933), also called probability matching, is an algorithm for MABs with binary rewards. The idea is to match the probability of choosing an action to its probability of being the best action. This means that the agent samples an estimated reward, $\hat{\theta_{a}}$, from each action's beta distribution and chooses the action with the highest such $\hat{\theta_{a}}$. The theoretical regret bound is logarithmic (Agrawal and Goyal, 2012).

## 3. Current Use of Multi-Armed Bandits in Brain-Computer Interfaces

There is limited use of MABs in BCI systems today. The following subsections describe two applications which together are representative of the state-of-the art of MAB in BCI. The MAB formulations utilized in these applications are variants of the original MAB problem formulation previously explained.

### 3.1. One Button BCI—Improving Calibration

Fruitet et al. (2012) have a BCI system with one button that the user can press by Motor Imagery (MI) movements, e.g., imagining moving the right hand (Pfurtscheller and Neuper, 2010). Different motor imagery tasks are optimal for different users and might also differ between sessions. Fruitet et al. aim to improve the calibration of such systems by focusing data collection on MI high-performing tasks rather than collecting data for all MI tasks, as in uniform calibration. In their MAB problem formulation, the set of actions *K* correspond to the available MI tasks, the time-horizon *T* to the total number of data samples to collect, the action *a*_*t*_ to the MI task of the following data sample to collect, and the reward *r*_*a*_*t*__ to the classification rate of the corresponding MI task. The goal for MAB problems is to maximize the total reward, while the goal for Fruitet et al. is to maximize the classification rate of the optimal MI task. Despite the slight goal difference, the exploration vs. exploitation trade-off is the same, and Fruitet et al. have based their algorithm on the UCB algorithm. They report higher classification rates with their algorithm than the uniform calibration approach. In a follow-up paper (Fruitet et al., 2013), they try their algorithm in an online setting and proves it to be more efficient than the uniform calibration approach, confirming their findings in the first paper.

### 3.2. Multi-Armed Bandits in P300 Spellers—Real-Time Implementations

In the original setup for a P300 speller, the letters are arranged in a grid, and a P300 signal is elicited when the row/column with the target letter is highlighted (Rezeika et al., 2018; Riggins and Scott, 2020). In the paper Ma et al. (2021), they use Thompson sampling to shorten the time for finding the target letter by reducing the number of non-target row/column highlights. In their MAB problem formulation, the set of actions *K* correspond to the available stimuli groups of letters to highlight, the action *a*_*t*_ to the next group, and the reward *r*_*a*_*t*__ (being 0 or 1) to whether the selected group contained the target letter or not. The actions' rewards follows a beta distribution where ${\hat{\theta }}_{a}$ represents the probability of the action's corresponding stimuli group containing the target letter. Their algorithm selects and evaluates multiple actions in each iteration, in contrast to classical MAB algorithms that select one action at each step. They use a pre-defined stopping criterion rather than a fixed time-horizon *T*. They conclude that the use of MABs improve the performance of the BCI system.

There are multiple variants of MABs in P300 spellers, e.g., Koçanaoğulları et al. (2018) and Guo and Huang (2021). The MAB problem formulation in Koçanaoğulları et al. (2018) is similar to Ma et al. (2021) (above), but Koçanaoğullari et al. additionally include language models as a priori information for the MAB algorithm. In Guo and Huang (2021), the agent uses a variant of the UCB algorithm which interprets EEG signals as contextual information when choosing actions. Only two actions with a binary reward *r*_*a*_*t*__ are available at each time step (the set of *K* is two actions), respectively, representing if the EEG signal had a P300 component or not.

## 4. Discussion of Future Use of Multi-Armed Bandits in Brain-Computer Interfaces

There are many promising uses for MABs in BCI systems. Here, we present some directions for future research and draw parallels to the MAB variants in Table 1. It is worth noting that mainly the original MAB formulation has been investigated in the context of BCI in literature, as is evident from the previous section.

### 4.1. Attention Selection

Adaptive hearing aids are currently being developed by many world-leading hearing aid companies. Often, adaptation is based on EEG measurements of the user's brain activity, deciphering their experienced hearing comfort. Hence, the adaptive hearing aid is in fact a BCI system, e.g., aiming to identify and aid the user in listening to an attended sound source (Alickovic et al., 2019).

In a MAB formulation of BCI based attention steering, each action corresponds to the hearing aid aiding the user in listening a surrounding sound source. The reward for each action should reflect the user's satisfaction with the hearing aid's selected sound source and could be measured from EEG data as Error potentials (ErrP) (Abiri et al., 2019), or the overall mental state (Krol et al., 2018). The MAB problem can be formulated in a few different ways based on different assumptions:

- Within a limited time, the surrounding sound sources are the same, and the user keeps the same interest in them. Hence, the reward for each action is stationary, analogous to the original MAB formulation.
- The user can change their preferred sound source at any time, which can be modeled with non-stationary rewards, such as a switching bandit formulation. One can assume as in Hartland et al. (2007) that it is only the best action that has a change in the reward, which means that the user can only lose interest in the target source, rather than hearing something else that gains their interest.
- Another approach would be to assume that sound sources can appear and disappear more or less randomly, which could be viewed as a mortal bandit problem as in Chakrabarti et al. (2008).

### 4.2. Data for Transfer Learning

A problem for BCI systems is the long calibration time due to the need for diverse data. Using data from previous sessions or persons and using transfer learning to adapt the old data to the current session is one solution (Lotte, 2015). To find relevant data, one can among other approaches use tensor decomposition (Jeng et al., 2021), Riemannian geometry (Khazem et al., 2021), or a generic machine learning model (Jin et al., 2020). In Gutiérrez et al. (2017), they use the classic MAB problem to find clusters of data in a big medical data set which increases the classification accuracy. The set of actions *K*, corresponds to the clusters, and the reward *r*_*a*_*t*__ mirrors the classification accuracy when using training data from the selected cluster. A similar setup could be used for transfer learning with BCI data.

### 4.3. Optimal Calibration Data

Another solution to the problem with calibration time (Lotte, 2015) is to collect calibration data cleverly. Instead of collecting from all classes, as in uniform calibration, data could be collected from the class that would improve the classification accuracy the most. Finding the optimal class could be formulated as a MAB problem where the set of actions *K* represent the available classes, and the reward *r*_*a*_*t*__ the gain in classification accuracy. Non-stationary rewards are a challenge in this setup since they will change with the current classification performance. Compared to the “one button BCI” described in Section 3.1, the aim here is to have a “multi-button BCI system” using all classes for control, while the “One button BCI system” aims to find a single optimal class and solely use that one for control (Fruitet et al., 2012, 2013). Another application would be for choosing the stimulation frequencies in a SSVEP-BCI.

### 4.4. Best Stopping Time

Another interesting aspect of the calibration phase in BCI systems raised in Fruitet et al. (2012) is to find the best *stopping time*. This means that the MAB agent stops taking actions before reaching the time horizon *T*. Stopping time is discussed in Lattimore and Szepesvári (2020a) for several algorithms. For the BCI system, this means that the calibration phase automatically stops, e.g., when the optimal class has been found for the task or when no further improvements to the classification can be made.

## 5. Getting Started With Multi-Armed Bandits in BCI Research

For most popular programming languages one can find examples of MABs (Github, 2022). Among other ready to use packages are: “SymPyBandits” for Python (Besson, 2018), “Bandits” for Julia (Celles et al., 2020), and “Contextual” for R (van Emden and Kruijswijk, 2020). None of these packages are aimed at MABs in BCIs. Hence, we provide a pedagogical Python script for MAB novices that can act as a starting point for future BCI research, inspired by Fruitet et al. (2012): https://gitlab.control.lth.se/FridaH/mab_for_bci-public/-/tree/main/MAB_for_BCI_1.

## 6. Conclusion

Multi-armed bandits (MABs) have been used successfully in many fields, yet few applications for Brain-Computer Interfaces (BCIs) exist. Firstly, this review summarizes MABs to the BCI community. Common algorithms to solve the classic MAB problem with stationary rewards include the ϵ-greedy policy, the UCB algorithm, and Thompson sampling, all with the aim to balance the trade-off between exploration and exploitation of available actions. Secondly, the review highlights current research that interprets and solves BCI problems as MAB problems, prominently occurring in calibration optimization and real-time implementations of BCI systems. Finally, some suggestions are provided on promising further research directions in the intersection of MABs and BCIs.

## Author Contributions

FH wrote the first draft of the manuscript. All authors have contributed to the conceptualization of the manuscript, manuscript revision, read, and approved the submitted version.

## Funding

This work was partially supported by the Wallenberg AI, Autonomous Systems and Software Program (WASP) funded by the Knut and Alice Wallenberg Foundation. All authors are also members of the ELLIIT Strategic Research Area.

## Conflict of Interest

The authors declare that the research was conducted in the absence of any commercial or financial relationships that could be construed as a potential conflict of interest.

## Publisher's Note

All claims expressed in this article are solely those of the authors and do not necessarily represent those of their affiliated organizations, or those of the publisher, the editors and the reviewers. Any product that may be evaluated in this article, or claim that may be made by its manufacturer, is not guaranteed or endorsed by the publisher.
